# Branchial cysts within the parotid salivary gland

**DOI:** 10.1186/1758-3284-4-24

**Published:** 2012-05-18

**Authors:** Tahwinder Upile, Waseem Jerjes, Mohammed Al-Khawalde, Panagiotis Kafas, Steve Frampton, Angela Gray, Bruce Addis, Ann Sandison, Nimesh Patel, Holger Sudhoff, Hani Radhi

**Affiliations:** 1 Department of Head and Neck Surgery, Chase Farm & Barnet NHS Trust, Enfield, UK; 2 Head & Neck Unit, University College London Hospital, London, UK; 3 ENT Department, Southampton General Hospitals, Southampton, UK; 4 UCL Department of Surgery, University College London, London, UK; 5 Oral and Maxillofacial Surgery Unit, AL-Mustansirya University’s, Baghdad, Iraq; 6 Department of Surgery, School of Dentistry, Al-Yarmouk University College, Baghdad, Iraq; 7 Oral and Maxillofacial Surgery Unit, Royal Medical Services, Amman, Jordan; 8 Department of Oral Surgery and Radiology, School of Dentistry, Aristotle University, Thessalonica, Greece; 9 Department of Pathology, Southampton General Hospital, Southampton, UK; 10 Department of Pathology, Charring Cross Hospital, London, UK; 11 Department of Otolaryngology, Head and Neck Surgery, Academic Teaching Hospital of University of Münster, Bielefeld, Germany

## Abstract

Cystic lesions within the parotid gland are uncommon and clinically they are frequently misdiagnosed as tumours. Many theories have been proposed as to their embryological origin. A 20-year retrospective review was undertaken of all pathological codes (SNOMED) of all of patients presenting with any parotid lesions requiring surgery. After analysis seven subjects were found to have histopathologically proven parotid branchial cysts in the absence of HIV infection and those patients are the aim of this review. Four of the most common embryological theories are also discussed with regard to these cases, as are their management.

## Introduction

Hunczowski described the first branchial cyst in 1789 [1]; however, the first surgical treatment of a branchial cyst was reported by Langenbeck in 1859 [2]. The first branchial cyst of the parotid gland was described by Hildebrandt in 1895 (this was at a time when HIV infection was unknown) [3]. The definition and origin of a 'branchial cyst' remains controversial [4,5]. It has been postulated that the cyst represents the remains of pharyngeal pouches or clefts [6,7].

Branchial (also called lymphoepithelial) cysts are uncommon findings in the oral cavity, major salivary glands, cervical lymph nodes, tonsils, thyroid gland, juxtabronchial and pancreas [8,9].They are often multicentric and may be unilateral or bilateral [10,11].

In the mid-1980’s, the association between branchial cyst of the parotid gland and HIV infection was first reported [12]. Since then, once the diagnosis of a branchial cyst is established, HIV testing is recommended, as it can be the first presentation of HIV infection [13].The incidence of branchial cysts is about 3-6 % in HIV-positive adults and 1-10 % in HIV-positive children [14,15]. They appear to be most common during the early phases of HIV infection [16].

Parotid branchial cysts are common in the third decade of life with a mean age of 44 years and a male to female ratio of 3:1 [17]. Branchial cysts can occur within the lymph nodes in the parotid gland and on the surface of the gland [10]. The cysts appear to be painless, slow-growing, firm, elastic and fluctuant masses and may vary in size from 0.5 cm to 5 cm in diameter [18]. In most of the cases the superficial lobe of the parotid gland is involved [19].

The cyst wall is usually lined by stratified squamous epithelium, pseudostratified columnar epithelium or a combination, with varying amounts of sub-epithelial lymphoid tissue in the form of diffuse bands, or follicles with germinal centres [20,21]. A benign parotid tumour is the commonest clinical diagnosis of a parotid mass, which results in superficial parotidectomy with general complete surgical excision and little recurrence [17]. When the nature of the swelling has been predetermined, careful enucleation of the cyst has been successfully performed [22].

The aim of this study was to review common features of parotid gland branchial cysts in the absence of HIV infection.

## Patients and methods

A 20 year retrospective histopathological review was undertaken of all pathological codes (SNOMED) of patients presenting to the central pathology services with any parotid lesions requiring surgery for excision. After analysis seven subjects were found to have histopathologically proven parotid branchial cysts, those patients are the subject of this review. The patients were HIV negative from serum testing at the time, (Table 1). Details were taken from pathology requests and case notes.

**Table 1:** Clinical details of 7 patients with parotid gland branchial cysts

Case	Age	Sex	Size (cm)	Location	Progression	FN involvement	↑size with infection	Pain
1	79	F	2*2*1	L lower pole	10 weeks	No	No	No
2	62	F	3*2*1	L lower pole	6 weeks	No	No	No
3	88	F	1.5*1*15	R lower pole	4 weeks	No	No	No
4	37	M	2*3*2	L lower pole	3 weeks	No	Yes	Yes
5	63	M	25*2*2.5	R lower pole	12 weeks	Yes	Yes	No
6	68	F	2.3*1.3	L intra parotid	8 weeks	No	No	No
7	35	F	2.0 diameter	R superficial lobe	4 weeks	No	Yes	Yes

Our unit’s current approach to any head & neck lesion involves ‘triple’ diagnosis by a combination of clinical (including endoscopy), ultrasound and fine needle aspiration perhaps augmented by radiological imaging including CT or MRI scanning. Obviously in this retrospective review collecting cases from over two decades it is unreasonable to assume that this diagnostic paradigm would be followed in every case. Review of the clinical and operative notes was performed. In all cases it was found that intraoperative facial nerve stimulators and or monitoring were used. The histological diagnosis was confirmed and common pathological changes correlated.

## Results

**Figure 1:**
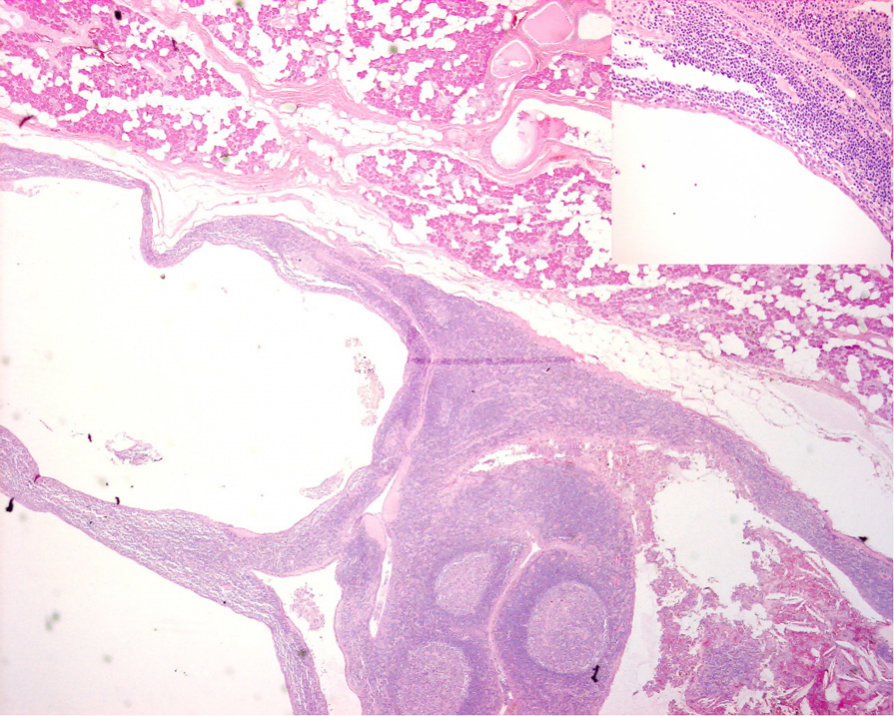
**This is a low power view of a H&E stained slide showing normal parotid gland (salivary tissue) with serous acini in the upper half of the field adjacent to multi-loculated lesional tissue with cystic spaces lined by lymphoid tissue in which there is florid lymphoid hyperplasia with prominent germinal centres.** The lumen of the cyst on the right contains haemorrhagic and keratinous debris including inflammatory cells and cholesterol clefts. The Inset (top right corner) is a medium power view of a H&E stained slide showing the epithelium lining the cyst wall which is mostly flattened squamous in type, showing the close relationship with the lymphoid stroma, as well as focal infiltration of lymphocytes into the epithelium.

**Figure 2:**
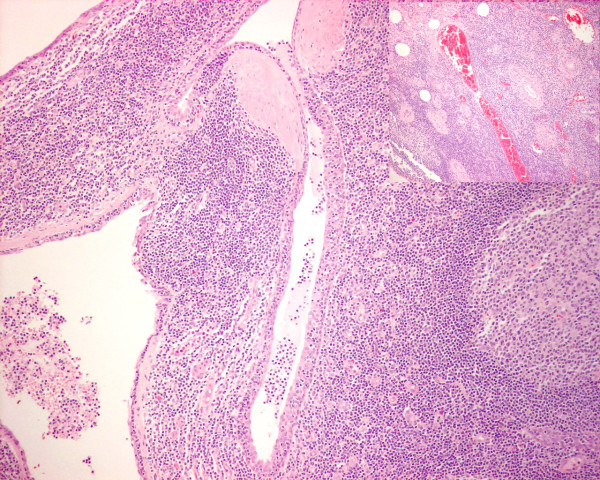
**This is a low power view of lesional tissue showing cystic spaces lined by squamous type epithelium with lymphoid tissue including a germinal centre on the right.** These cysts show a mixture of squamous and ductal type epithelial lining with prominent infiltration by small lymphocytes. The Inset (top right corner) is low power view of a H&E stained slide showing ductal structures surrounded by blood vessels with abundant lymphoid tissue in the adjacent stroma. Scattered small islands of epithelium are identified in the lymphoid stroma. These represent branchial pouch-derived inclusions which proliferate to form cysts under the influence of growth factors produced by the hyperplastic lymphoid tissue. In line with the lymph node inclusion theory, some of these consist of pink staining oncocytic epithelium of the type as seen in Warthin’s tumours.F

**Figure 3:**
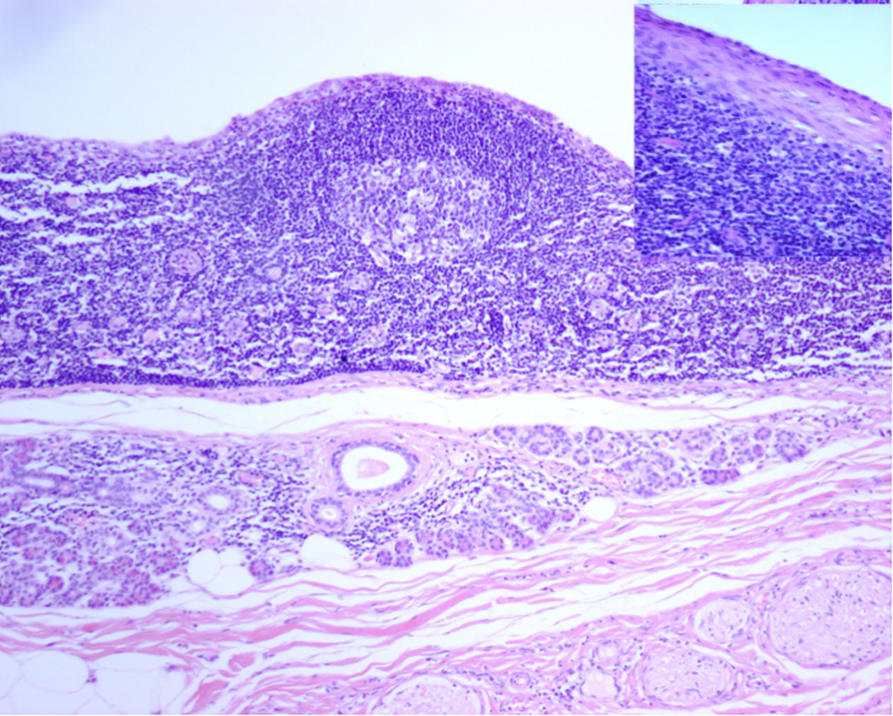
**The section shows neurovascular tissue at the top, adjacent to normal parotid salivary gland which in turn lies a cyst wall lined by bland epithelial cells with lymphoid tissue including a germinal centre in the wall.** The Inset (top right corner) is medium power view of a cyst wall lined by bland squamous epithelium with abundant mixed lymphoid cells in the wall.

**Figure 4:**
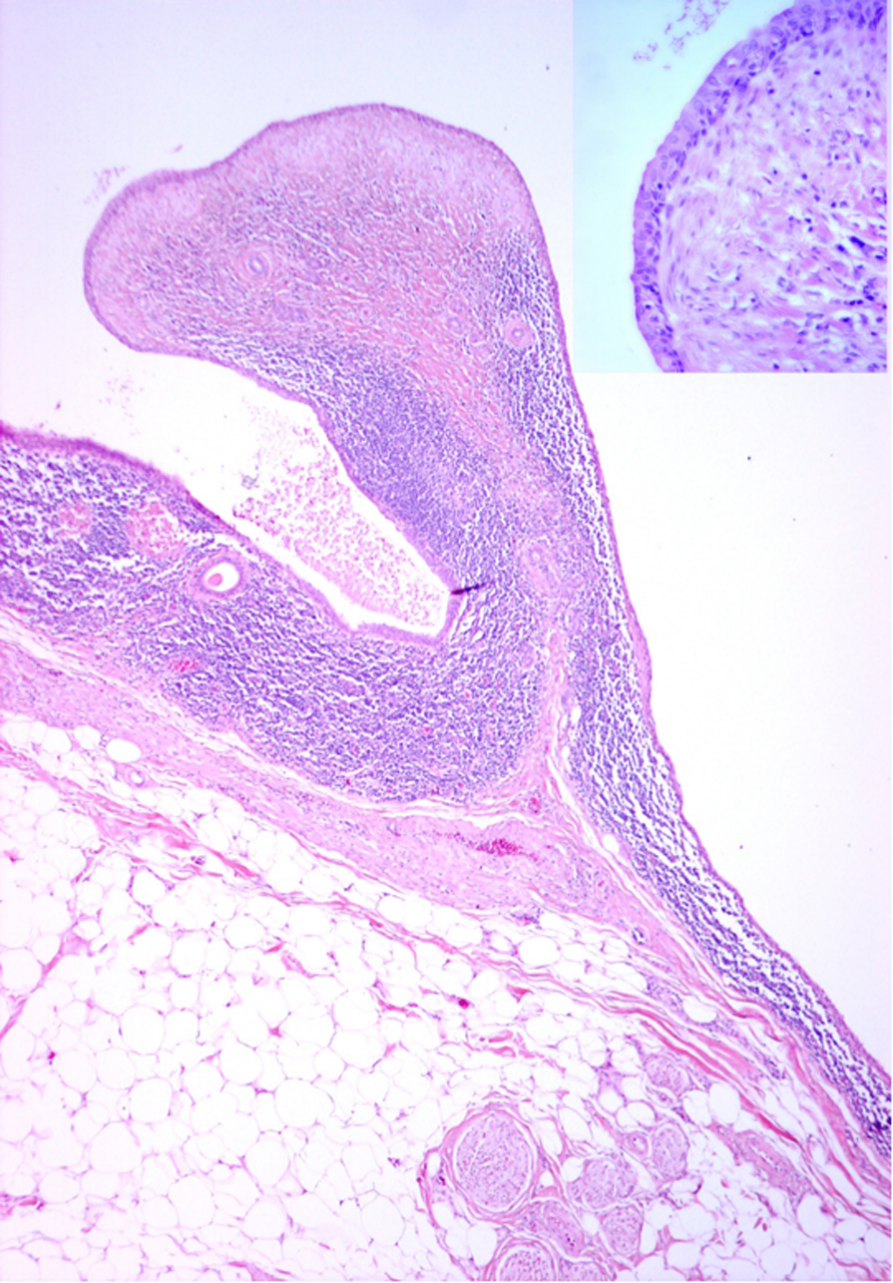
**This shows vascular adipose tissue and nerve bundles underlying a cystic structure lined by bland squamous epithelium with lymphoid tissue in the wall.** Also in the wall of the cyst is a ductal structure. The Inset (top right corner) shows a high power view of a structure lined by bland epithelial cells and fibrous tissue in the stroma.

The average age of presentation of these subjects was 61.7 years, five subjects were females and five cysts were located within the lower pole of the parotid gland. The average duration of acute symptoms was 6.7 weeks; three patients, (cases 4, 5 and 7), having an increase in the size of their branchial cysts with infection; two of those patients, (cases 4 and 7) experienced concurrent severe pain (Table 1). In all cases the preoperative diagnosis included a tumour and a superficial parotidectomy was performed. Histology in each case revealed features of branchial cysts (Table 2), (Figures 1, 2, 3, and 4).

**Table 2:** Preoperative diagnosis and pathology of 7 patients with parotid gland branchial cysts

Case	Pre-operative diagnosis	Pathology
1	? tumour	Cyst lined by stratified squamous epithelium with lymphoid material
2	? tumour	Cyst lined by stratified squamous epithelium with lymphoid material
3	? tumour	Cyst lined by stratified squamous epithelium with lymphoid material
	?lymph node	
4	?tumour	Cyst lined by stratified squamous epithelium with lymphoid material
	?pharapharyngeal abscess	
5	? tumour	Cyst lined by stratified squamous epithelium with lymphoid material and pseudostratified columnar epithelium and fibrous wall
	?cyst	
	?residual lymphangioma	
6	? tumour	Multilocular cystic structure filled with treacle-like fluid. Intraparotid lymph node with epithelial cysts, lined by mainly ductal type epithelium with foci of oncocytic change. Background parotid normal. No evidence of malignant change
	?benign pathology	
	?lymph node	
	?preparotid lipoma	
7	?tumour	Thin walled cystic nodule 22 mm diameter. Contains brown mucoid material. Cyst lined by attenuated ductal epithelium with areas of squamous metaplasia. The wall consists of hyperplastic lymphoid tissue. Adjacent parotid shows small foci of chronic inflammation, some related to ducts some with features of lymphoepithelial sialadenitis. No evidence of malignancy
	?lymphoepithelioid cyst	

All patients were shown to be HIV negative on serum testing at the time. The possibility of false negative cannot be excluded however review of notes did not indicate any significant illnesses (HIV disease defining) in the interval of the study.

## Discussion

Branchial cysts of the parotid gland are uncommon [23]. The aim of this case series and review was to show that they can occur in the absence of HIV infection. Their embryological origin still remains controversial and many theories have been suggested [17]. Just as with i.e. Kaposi’s sarcoma, the prevalence of branchial cysts of the parotid has increased with HIV infection. The differentiation of a branchial cysts and cystic degeneration within sqamous cell carcinomatous metastases to a lymph node must always be borne in mind when interpreting FNA, imaging and in planning the surgical approach.

Four of the most common theories are outlined as the following:

***The branchial apparatus remnant theory*** suggests that the lining epithelium of the branchial cyst is derived from branchial cleft ectoderm (shown as stratified squamous epithelium in cases 1 to 4) or branchial arch/pouch endoderm (pseudostratified columnar epithelium) or both epithelial types as in case 5 of a long-standing cyst [6,7]. They remain dormant until an external stimulus causes cystic proliferation [6,7,24-26]. This is very similar to a study of branchial cysts which found that 18 % of such cysts contained a lining of pseudostratified columnar epithelium not known to normally arise in the parotid gland [25]. In our series the average age of presentation was 61.7 years, which is very much later than would be expected (i.e. third decade) from the usual congenital branchial cysts. Remnants of the first branchial clefts occur along an imaginary line extending from the auditory canal behind and below the angle of the mandible to its mid-point. The second branchial cleft remnants are found anywhere along a line extending from the tonsillar fossa down to a point on the lower one third of the anterior border of sternocleidomastoid [27]. In this review five of the parotid 'branchial cysts' were located in the lower pole of the gland and hence may have been derived from either first or second clefts. The second branchial arch is the most likely source of most branchial cysts except from intra-parotid cysts which may be accounted for by first arch anomalies [28]. These first branchial cleft anomalies are rare accounting for between 1-8 % of all branchial apparatus defects and are one sixth as common as second cleft abnormalities [28]. They are most likely to affect the parotid gland resulting in a mass or inflammation and 68 % occur as a cyst [29].

***The Cervical Sinus Theory*** similarly suggests that these parotid branchial cysts represent the remains of the Cervical Sinus which is formed when the second branchial arch grows caudally to meet the fifth [30,31]. This may have been the aetiology of case 5 but this is doubtful.

***The Thymopharyngeal Duct Theory*** was initially proposed by Wenglowski in 1913 [32] and later by Meyer, McNealy in 1932 [33,34]. It suggests that these cysts are the remnants of the original connection between the thymus and the third branchial pouch from which it derives. This is unlikely from the results of our histopathological review which failed to show any histological evidence of this tissue type [29,31].

***The Lymph Node Inclusion Theory*** suggests that these cysts are a result of cystic alteration of epithelium trapped in the cervical lymph nodes [23,30]. It proposes that this is more likely in the upper one third of the neck where parotid epithelial inclusions are most likely to occur [35]. This close association of salivary gland and lymphoid tissue is supported by the fact that the fetal unencapsulated parotid is intimately associated with the developing parotid and cervical lymph nodes [17,30,35]. Further support lent by the observation that salivary tumours also arise in epithelium with primary Warthin's Tumour occasionally found to arise in cervical lymph nodes [36]. Again in support of this theory all the cysts examined in this series had lymphoid material present within their walls, especially in cases 6,7. It may be these cysts are a combination of the heterogeneous branchial cleft Work type II (occasionally type I) anomalies containing lymph node epithelial inclusions to varying degrees.

The facial nerve involvement evident in case 5, may be the result of previous surgery, concurrent anomaly of facial nerve distribution [37], or even the result of local inflammation around the cyst closely related to the nerve branches [38]. The nerve, when involved, appeared to be within the walls of the cyst and required careful dissection. In no case was the nerve sacrificed. All cases had perioperative facial nerve stimulation or monitoring.

The three cases (4, 5 and 7) of an increase in the size of the branchial cysts associated with infection, has been recognised as a characteristic of the condition in reports of both fluctuations in the sizes of some branchial cysts, in association with local inflammatory conditions and occasionally pain, as in cases 4 and 7 [28]. It is suggested that incision and drainage are indicated in these cases where abscesses have developed with complete surgical excision after resolution of the infection. In case 5, previous inadequate incision may have resulted in its recurrence and increased risk of and subsequent infection and fluctuation in size. It may be case 5 represents a differing subtype of parotid gland branchial cyst, perhaps Work type II cysts, whilst cases 1–4, 6, 7 represent type I cysts [6,7,24-26].

A number of preoperative investigations may be useful in establishing the diagnosis [18]. A computed tomography (CT) scan is a useful tool in distinguishing solid from cystic lesions of the parotid gland although it sometimes carries a risk of false positive results [39]. An ultrasound scan has also been proved to be a rapid, inexpensive and readily available tool in differentiating solid from cystic lesions [40,41]. Magnetic resonance imaging can also be helpful in distinguishing a branchial cyst from a tumour [17].The cysts appear to be dark on T1 sequence and bright on T2 sequence, reflecting the protein content of the fluid [42]. However, it must be remembered that parotid gland branchial cysts are rare and that the majority of parotid swellings will remain neoplastic [27].

The ultrasound appearances of HIV associated can range from a simple cyst to heterogenous mass and even completely solid lesions. These cystic lesions can have thin septa supplied with vascular pedicles. CT scanning appearances suggest hypodense lesions. While on MRI, their features are consistent with a low signal in T1 and high signal in T2, with signal characteristics of fluid. HIV parotid cysts are often

Fine needle aspiration (FNA) can be helpful in the diagnosis of parotid masses and to rule out malignancy, especially in HIV-positive patients [43]. Macroscopically it may reveal the presence of a yellow, watery and rarely turbid fluid which may or may not reaccumulate rapidly and can be indicative of a branchial cyst [44]. FNA smears [4] of branchial cysts can also microscopically reveal variable numbers of mature squamous cells, anucleate squames, cell debris, proteinaceous material, macrophages and lymphocytes with cystic fluid, occasionally having high amylase content [28,44]. We commend the ultrasound directed FNA of the cyst wall, alternatively complete aspiration of the cyst contents and then repeat FNA of the solid component s of the lesion may provide higher diagnostic yields. However, in the presence of inflammation (as in cases 4, 5 and 7) or neoplasm, immature squamous cells with increased nuclear to cytoplasmic ratio with nuclear hyperchromasia may be present [18]. In rare cases it may be impossible to distinguish a branchial cyst showing evidence of repair, from a metastatic carcinoma i.e. in a degenerated necrotic lymph node [4]. Hence excisional biopsy by superficial parotidectomy is the standard diagnostic and therapeutic intervention for such cysts [45]. If the diagnosis of a parotid branchial cyst is certain, then simple partial lateral superficial parotidectomy with preservation of the facial nerve is recommended, perhaps via a modified retroauricular incision [22]. Otherwise, a formal superficial parotidectomy with intraoperative facial nerve monitoring should be undertaken to prevent inadequate excision of a tumour and recurrence of the branchial cyst [6,7,24-26].

## Consent

Written informed consent was obtained from all of the patients for publication of these cases and any accompanying images.

## Competing interests

The authors declare that they have no competing interests.

## Authors’ contributions

TU, WJ, MA, PK, SF, AG, BA, AS, NP, HS, HR designed the study, carried out the literature research and manuscript preparation. TU, WJ, MA, PK, SF, AG, BA, AS, NP, HS, HR were responsible for critical revision of scientific content and manuscript preparation and review. All authors contributed to conception and design and approved the final version of the manuscript.
